# Growth and metabolic characterization of four lactic acid bacteria species isolated from rice beer prepared in Assam, India

**DOI:** 10.1099/acmi.0.000028

**Published:** 2019-05-29

**Authors:** Arup Jyoti Das, Manas Jyoti Das, Tatsuro Miyaji, Sankar Chandra Deka

**Affiliations:** 1 Department of Food Engineering and Technology, Tezpur University, Tezpur, Assam 784028, India; 2 Department of Materials and Life Science, Faculty of Science and Technology, Shizuoka Institute of Science and Technology, 2200-2 Toyosawa, Fukuroi, Shizuoka 437-8555, Japan

**Keywords:** rice beer, lactic acid bacteria, 16s rDNA, gut tolerance, adherence, antioxidant activity

## Abstract

Isolation and identification of lactic acid bacteria (LAB) from rice beer prepared in Assam, India was performed and their growth associated and functional properties were studied. LAB strains were identified as *Lactobacillus casei *, *Pediococcus pentosaceus *, *Lactobacillus pentosus* and *Lactobacillus plantarum* based on 16 s rRNA sequencing. Their growth characteristics at different pH, NaCl concentration, temperature and presence of carbohydrates were profiled. High tolerance against acid and bile salts was shown by all the strains, particularly *L. pentosus* TEZU174 and *P. pentosaceus* TEZU199 up to a pH of 1.5, and *L. pentosus* TEZU174 up to 14  % bile concentration. They were susceptible towards the common antibiotics, wherein erythromycin, chloramphenicol and linezolid were the most effective. The strains displayed antibiosis activity against *Escherichia coli* and *Staphylococcus aureus* and antioxidant activity in terms of resistance to H_2_O_2_, scavenging of ·OH and DPPH free radicals was also displayed, wherein *L. casei* TEZU374 and *P. pentosaceus* TEZU482 were the most effective with above 70  % scavenging activity. The strains displayed cellular aggregation and *L. casei* TEZU262 and *L. casei* TEZU309 were highly aggregated, which attained 100  % autoaggregation within a period of 5 h. High cell surface hydrophobicity was shown by *L. casei* TEZU309 towards xylene and chloroform, and *P. pentosaceus* TEZU427 towards ethyl acetate. The strains evinced good gut tolerance capacity, antioxidant activity and adherence properties, which are characteristics of probiotic bacteria and thus are candidates for therapeutic uses and also to be used as starter cultures.

## Introduction

In Asian countries, beer prepared from rice using traditional solid-state starters is a popular traditional alcoholic beverage [1, 2]. These are highly nutritious and functional due to the content of various proteins, sugars, vitamins, bioactive compounds and various organic acids [3, 4]. They are also a gluten-free alternative to conventional barley beer; the consumption of which is not safe for gluten-sensitive coeliac patients [5]. These beers are produced differently than conventional beers made from barley or wine made from grapes. The starters for fermentation are cake-shaped products made from wheat or grits mixed with medicinal plants, which allows the growth of various natural fungi, yeast and lactic acid bacteria (LAB) [3, 6, 7]. The final produce is obtained through a rough filtration of the fermented mash, and hence contains a high count of microbes involved in the fermentation process [3]. The moulds present in the starters produce α-amylase and amyloglucosidase that results in liquefaction and saccharification of rice starch thereby producing dextrins, maltose and glucose [1]. Here parallel fermentation occurs, i.e. progressive saccharification of starch by moulds and alcoholic fermentation of the liberated glucose by yeasts, thereby avoiding the exposure of yeast cells to high concentrations of sugar, and contributing to the high ethanol production [8]. The pH is acidic due to the organic acids such as lactic, citric and tartaric acids; together with acetoin they impart a sour taste and a pleasant flavour [5].

In East-Asia, lactic acid fermentation has been applied for various purposes such as to preserve perishable vegetables, prepare acidic dishes, to ensure the quality of rice wines, and to make plant-based beverages [9]. LAB are Gram-positive and usually catalase-negative bacteria, which comprise a wide range of genera and the most important ones are *Lactobacillus *, *Lactococcus *, *Enterococcus *, *Streptococcus *, *Pediococcus *, *Leuconostoc *, *Weissella *, *Carnobacterium *, *Tetragenococcus *, *Oenococcus* and *Vagococcus* [10, 11]. With over 60 species, *Lactobacillus* is a heterogeneous genus (ymol% G+C content ranging from 33 to 55) about one-third of which are strictly heterofermentative [10]. LAB has long been safely applied in the production of fermented foods and beverages. They occupy a central role in traditional starter cultures and are known to enhance the shelf life, and improve microbial safety, texture and sensory profile of the fermented product, mainly through the production of organic acids, ethanol, aroma compounds, bacteriocins, exopolysaccharides and several enzymes. They have been industrially used in the production of antimicrobial substances, sugar polymers, sweeteners, aromatic compounds, useful enzymes, or nutraceuticals, or as probiotics [12].

There are several potential health or nutritional benefits from some species of LAB, which includes improved nutritional value of foods, control of certain types of cancer and intestinal pathogens, control of serum cholesterol levels and improved lactose utilization. In order to utilize a specific benefit from LAB, it is necessary to consider not only the wide variation among species but also among the strains [13]. LAB are common components of probiotics, due to the fact that they are ‘generally regarded as safe’ as they have long been used in the manufacture of dairy foods and are desirable members of the intestinal microflora [14]. When probiotics are added to any food product, they should be able to survive in the product and become active when entering the consumers’ gastrointestinal tract [15]. Hence a study of various physiological and functional properties of LAB strains is essential to be carried out before they can be applied for any probiotic purpose. In this study the functional properties of some lactic acid bacteria isolated from rice beer prepared in Assam, India have been evaluated, with the purpose of establishing them as potential probiotic strains.

## Methods

### Collection of samples

The rice beer and starter cakes were collected from the households of different tribal communities of Assam. All the samples were collected in replicates in sterile bottles, and brought to the laboratory under refrigerated condition and stored at 4 °C until further analysis. The chemicals, solvents and microbial growth media were obtained from Sigma-Aldrich Corporation, USA and HiMedia, India. The test microbes were obtained from the Department of Food Engineering and Technology, Tezpur University, Assam.

### Isolation and identification of the isolates based on 16s rRNA sequencing

The LAB were isolated following the method described by Brown [16], by plating on MRS agar supplemented with CaCO_3_ and bromocresol purple indicator using a double-layer technique. The genomic DNA from 18 h old cultures were isolated and purified using a DNA purification kit (K0512, Thermo Scientific, EU) and the concentration was measured spectrophotometrically. For PCR amplification, the 16S rRNA universal primers set consisted of ‘27 f’ forward primer (5′-GAGAGTTTGATCCTGGCTCAG-3′) and ‘1495 r’ reverse primer (5′-CTACGGCTACCTTGTTACGA-3′). Amplification was carried out in a thermocycler (Mastercycler nexus gradient, Eppendorf, USA), with initial denaturation at 95 °C for 3 min, followed by 30 cycles of 94 °C (1 min), 55 °C (1 min), 72 °C (1 min), linked to 72 °C (10 min) and then to 5 °C. The amplified DNA was purified using a PCR product purification kit (GeneJET K0701, Fermantas, EU), and was separated by agarose gel electrophoresis. DNA sequencing was performed by Sanger method in an automated sequencer at the SciGenom Labs, Cochin, India. The organisms were identified with the help of the highest maximum identity score from the GenBank database using the blast tool, and the sequences were submitted to the National Center for Biotechnology Information (NCBI) for obtaining accession numbers.

The sequences of identified phylogenetic neighbours were aligned using ClustalW and a phylogenetic tree was constructed by the neighbour-joining method using mega 7.0.14 software [17]. The arbitrarily rooted tree was created from evolutionary distances using a maximum-likelihood approach and there were a total of 627 positions in the final dataset. The maximum likelihood method based on the Tamura–Nei model [18] was used to infer the evolutionary history. The branch lengths were measured in the number of substitutions per site and first, second, third and non-coding codon positions were included after eliminating all positions with gaps and missing data.

### General characteristics of the strains

A catalase test was performed using 3 % H_2_O_2_ and a test for the production of gas from glucose was performed on phenol red broth tubes containing inverted Durham's tubes [19]. A test for the production of ammonia from arginine was performed using Nessler's reagent [20]; gelatinase activity was tested with saturated ammonium sulfate solution [21] and haemolysis activity was determined on sterile blood agar [22].

### Growth characteristics at different pH, salt concentration and temperature

De Man, Rogosa and Sharpe (MRS) broth was adjusted to pH of 3.9, 9.6 and 7.0 with 1 N HCl and 10 % NaOH solution, and 6.5, 10 and 18 % salt concentration using NaCl. The LAB strains were inoculated and incubated in these media at 35 ^○^C for 48 h. To test the influence of temperature, the LAB cultures on MRS broth were grown at 10, 15 and 45 °C. The growth in all the tests was assessed spectrophotometrically at 600 nm [19].

### Carbohydrate fermentation tests

The LAB strains were grown on phenol red broth supplemented with glucose, lactose, maltose, sucrose, xylose, mannose, trehalose, galactose, raffinose, arabinose, salicin, sorbitol, dulcitol and cellobiose at 5 mg ml^−1^. Incubation was done at 37 °C for 48 h and the tubes were examined up to 48 h for evidence of acid production, indicated by the medium turning yellow [23].

### Test for acid and bile tolerance

For acid tolerance test, MRS broth was adjusted to pH 1.5, 2.0 and 7.0 using 1.0 N HCl and NaOH respectively, and inoculated with 18 h old LAB cultures at 10^8^ c.f.u. ml^−1^. Plating from each tube on MRS agar was done after an interval of 0, 1, 2, 3, 5, 6, 7, 8, 24 and 48 h after incubation at 37 °C. For bile tolerance test, 24 h old LAB cultures were inoculated in MRS broth supplemented with bile salts (1 to 14 %) at 10^8^ c.f.u. ml^−1^ and incubated at 37 °C, followed by plating on MRS agar after 24 h [24].

### Antibiotic susceptibility test

This was carried out using antibiotic discs, which were placed upon the antibiotic susceptibility test media Mueller−Hinton agar (MHA) plates, which were preinoculated with 48 h old cultures of the LAB strains. Incubation was done for 24 h at 37 °C under anaerobic condition by using an anaerobic gas pack system (LE012, Himedia, India), following which the inhibition zones were measured using an antibiotic zone scale [25]. The antibiotics used were ampicillin, chloramphenicol, ciprofloxacin, erythromycin, penicillin, gentamycin, kanamycin, rifampicin, streptomycin, tetracycline, vancomycin and linezolid.

### Antibiosis activity tests

The antibacterial activity against *Escherichia coli* MTCC 40 and *Staphylococcus aureus* MTCC 3160 was tested by employing the agar spot-on-lawn test of Schillinger and Lucke [26]. The LAB cultures were first spot inoculated on MRS agar plates and grown for 48 h under anaerobic condition. Then 0.25 ml culture of the indicator bacteria (18 h old) was mixed with 9 ml of antibiotic susceptibility test media (0.7 % agar) at 50 °C and poured over the spotted plates. The plates were incubated for 24 h at 37 °C, following which the inhibition zones were observed and expressed as the difference in diameter between the total zone of inhibition and the LAB growth spot. For antifungal activity, 2 cm long 48 h old streaks of the LAB cultures on MRS agar plates were overlaid with 10 ml of malt extract soft agar (0.7 %) containing 10^4^ fungal (*Aspergillus niger* MTCC 281) spores/ml and incubated at 30 °C for 48 h. Clear zones of inhibition surrounding the bacterial streaks indicated inhibition [27].

### Tests for antioxidant activities

These were performed according to Li *et al*. [28]. To test the resistance to H_2_O_2_, 1 % (v/v) overnight cultures of LAB were inoculated into MRS broth containing 0.4, 0.7 or 1.0 mM hydrogen peroxide and incubated at 37 °C for 8 h. The cell growth was measured spectrophotometrically at 600 nm. For ·OH and DPPH radical scavenging activities, the 18 h old LAB cultures were resuspended in deionized water to approximately 10^8^ c.f.u. ml^−1^. For ·OH scavenging activity, the cell suspension was incubated with 1 mg ml^−1^ lysozyme at 37 °C for 30 min followed by ultrasonic disruption for five 1 min intervals in an ice bath. This was centrifuged and 1 ml of the intracellular cell-free supernatant was taken and incubated with 1 ml of 0.435 mM brilliant green solution, 2 ml of 0.5 mM FeSO_4_ solution and 1.5 ml of 3.0%, w/v H_2_O_2_for 20 min, and the scavenging activity was expressed according to equation 1.

(1) $$Scavenging\;activity\;\left(\%\right)=\left[\left(A_{S}-A_{o}\right)÷\left(A-A_{O}\right)\right]\times 100,$$

where *A*_*S*_ is the absorbance in the presence of the cellular extract, *A*_*0*_ is the absorbance of the control in the absence of the cellular extract, and *A* is the absorbance in the absence the cellular extract, FeSO_4_ and H_2_O_2_, all read at 624 nm.

For DPPH radical scavenging activity, 2 ml of the cell suspension was mixed with 4 ml of 0.05 mM ethanolic DPPH solution and incubated for 30 min. The absorbance (*A*) of the resulting solution was measured at 517 nm and scavenging activity was expressed according to equation 2. The controls included deionied water and DPPH solution.

(2) $$Scavenging\;activity\left(\%\right)=\;\left[1-\left(A_{Sample}-A_{Control}\right)÷A_{Control}\right]\times 100.$$

### Test for cellular autoaggregation

The LAB were grown for 18 h and resuspended in PBS to approximately 10^8^ c.f.u. ml^−1^. This suspension was mixed by vortexing for 10 s and every hour, 0.1 ml of the upper suspension was mixed with 3.9 ml of PBS and absorbance was measured at 600 nm. This was done for 5 h and the autoaggregation percentage was expressed according to equation 3, where *A_t_*=absorbance at 1, 2, 3, 4 or 5 h and *A_0_*=absorbance at 0 h [29].

(3) $$Autoaggregation\;\left(\%\right)=100\times \left(A_{O}-A_{t}\right)÷A_{O}.$$

### Test for cell surface hydrophobicity

This was determined by using the microbial adhesion to solvents (MATS) assay [30]. The LAB cultures (18 h old) were resuspended in phosphate-urea-magnesium (PUM) buffer to a count of approximately 10^8^ c.f.u. ml^−1^ and mixed thoroughly with xylene in 1:3 ratio. This was incubated at 37 °C for 70 min and absorbance of the aqueous phase was taken at 600 nm. MATS was calculated according to equation 4, where *A_f_* is the absorbance of aqueous phase after mixing and *A_i_* denotes the phase separations relative to that of original suspension. Similarly MATS towards chloroform and ethyl acetate was calculated, which are regarded as a measure of electron donor (basic) and electron acceptor (acidic) characteristics, respectively [31].

(4) $$MATS\;\left(\%\right)=100\times \left(A_{f}-A_{i}\right)÷A_{i}.$$

## Results

### The LAB strains identified in the starter cakes

The different identified species of LAB isolated and identified from various rice beer starter cakes used by different communities in Assam are shown in Table 1. From the various starters, three strains of *L. casei *, one strain of *L. pentosus *, one strain of *L. plantarum* and eight strains of *P. pentosaceus* were identified.

**Table 1.** Identified strains and their NCBI accession numbers

Rice beer/starter cake name, Community	Organism name	Accession Number
*Umhu,* Dimasa	* L. casei* TEZU309	KR781616
*Judima*, Dimasa	* L. pentosus* TEZU174	KR781611
*Thap*, Karbi	* L. casei* TEZU468	KT273335
*Hor-alank*, Karbi	* P. pentosaceus* TEZU199	KR781612
*Perok-kushi*, Deori	* P. pentosaceus* TEZU451	KT273336
*Sujen*, Deori	* P. pentosaceus* TEZU213	KR781617
*Mod-pitha*, Ahom	* L. plantarum* TEZU272	KT273347
*Xajpani*, Ahom	* P. pentosaceus* TEZU427	KT273337
*Apop-pitha*, Mising	* P. pentosaceus* TEZU481	KT273331
*Apop-pitha*, Mising	* P. pentosaceus* TEZU482	KT273330
Apop-pitha, Mising	* P. pentosaceus* TEZU486	KT273328
*Apong*, Mising	* L. casei* TEZU262	KR781613
*Amou*, Bodo	* P. pentosaceus* TEZU410	KT273338
*Jou-bishi,* Bodo	* L. casei* TEZU374	KT273339

**Fig. 1.**
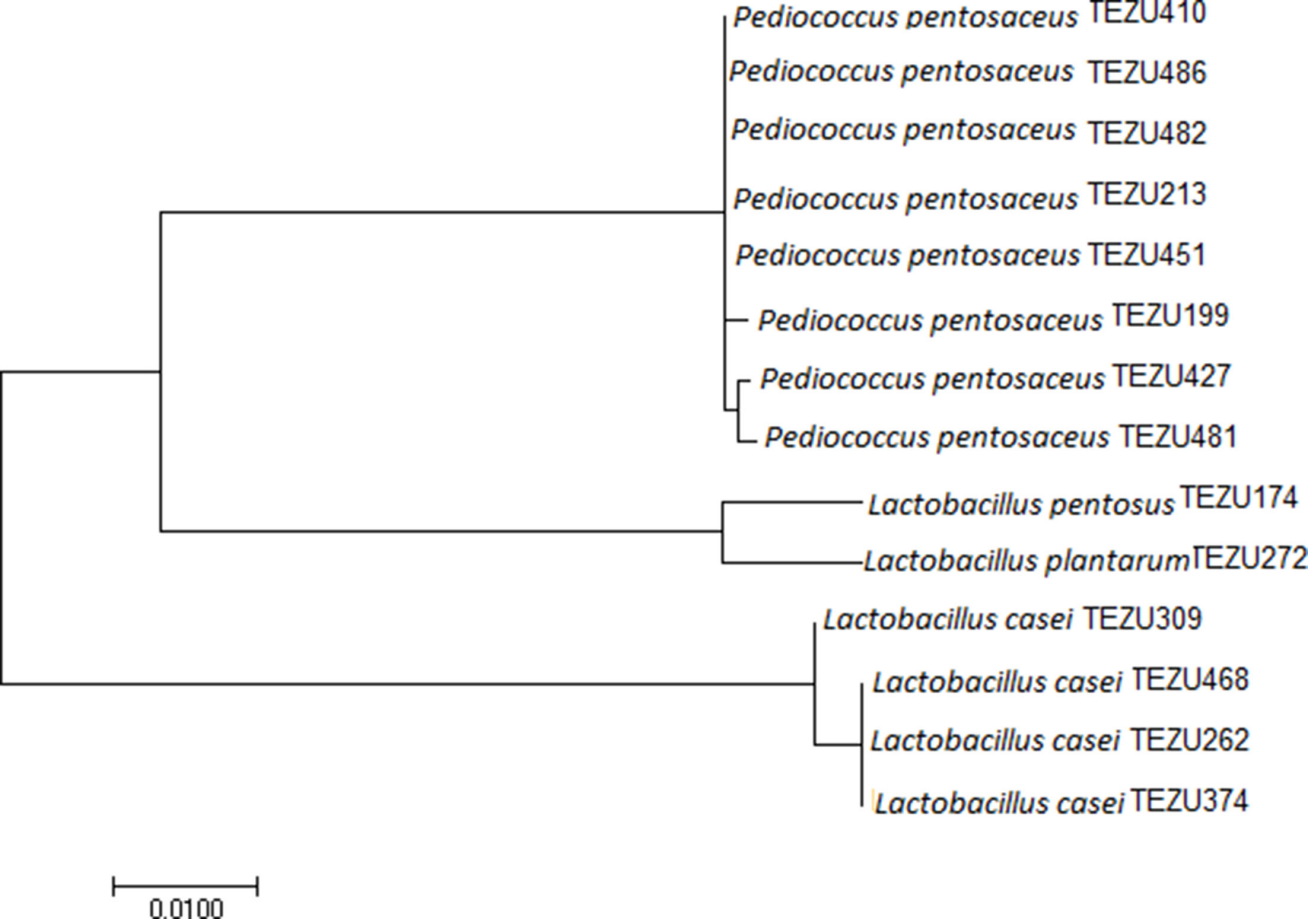
Phylogenetic tree of the LAB strains obtained by molecular phylogenetic analysis through the maximum likelihood method.

The phylogenetic tree with the highest log likelihood (−1399.4482) was selected (Fig. 1). The analysis involved 14 nucleotide sequences. The phylogenetic tree was constructed based on 500 samplings in which the neighbour-joining and BioNJ algorithms [18] were applied to a matrix of pairwise distances estimated using the maximum composite likelihood (MCL) approach. The topology with superior log likelihood value was selected to obtain the initial trees for the heuristic search. Close association was seen between *P. pentosaceus* TU427 and *P. pentosaceus* TU481, which were related to the rest of the *P. pentosaceus* strains in the next branch. Relation was also seen between *L. pentosus* TU174 and *L. plantarum* TU272. The remaining *L. casei* strains were also closely related.

### General characteristics of the LAB strains

All the strains were found to be positive for the production of catalase and negative for the production of gas from glucose, production of ammonia from arginine, gelatin hydrolysis and haemolytic activity.

### Growth at different pH, NaCl concentration and temperature

The results for accessing the growth of the LAB strains under these conditions are shown in Table 2. It was observed that all the strains could grow at pH 7. However, at pH 9.6, *L. casei* TEZU309, *L. pentosus* TEZU174 and *L. plantarum* TEZU272 could not grow. At low pH of 3.9, *L. plantarum* TEZU272, *P. pentosaceus* TEZU427, *P. pentosaceus* TEZU482, *P. pentosaceus* TEZU486, *L. casei* TEZU262 and *L. casei* TEZU374 showed some growth, while the rest of the strains could not grow. Most of the strains except *L. casei* TEZU309 could grow at 6.50 % NaCl, whereas at 10 % NaCl *L. pentosus* TEZU174, *L. pentosus* TEZU174, *P. pentosaceus* TEZU213, *L. plantarum* TEZU272, *P. pentosaceus* TEZU481 and *P. pentosaceus* TEZU410 could not grow. At the highest concentration of 18 % NaCl only the strain *P. pentosaceus* TEZU427 exhibited some growth. At 10 °C, most of the strains showed poor growth, and six of the strains could not grow. At 15 °C only the strain *P. pentosaceus* TEZU410 could not grow. Whereas, at 45 °C the strains *L. casei* TEZU309, *L. casei* TEZU468 and *P. pentosaceus* TEZU451 displayed proper growth.

**Table 2.** Growth characteristics of the strains at different pH, NaCl concentration and temperature

Strain name	pH			NaCl			Temperature		
	3.9	7	9.6	6.50 %	10 %	18 %	10 °C	15 °C	45 °C
* L. casei* TEZU309	−	++	−	−	−	−	+(w)	+	++
* L. pentosus* TEZU174	−	++	−	+	−	−	−	+	−
* L. casei* TEZU468	−	++	++	+(w)	+(w)	−	+(w)	++	++
* P. pentosaceus* TEZU199	−	++	++	++	+	−	−	+	−
* P. pentosaceus* TEZU451	−	++	+	+(w)	+(w)	−	+(w)	++	++
* P. pentosaceus* TEZU213	−	++	++	++	−	−	−	+	−
*L .plantarum* TEZU272	+(w)	++	−	++	−	−	−	+(w)	−
* P. pentosaceus* TEZU427	+(w)	++	++	++	+(w)	+(w)	+(w)	+(w)	+
* P. pentosaceus* TEZU481	−	++	+	+(w)	−	−	+(w)	++	+
* P. pentosaceus* TEZU482	+(w)	++	+	+(w)	+(w)	−	+(w)	++	+
* P. pentosaceus* TEZU486	+(w)	++	+	+(w)	+(w)	−	+(w)	++	+
* L. casei* TEZU262	+	++	++	++	+	−	−	+(w)	−
* P. pentosaceus* TEZU410	−	++	+	+	−	−	−	−	−
* L. casei* TEZU374	+(w)	++	++	+	+(w)	−	+(w)	+	++

*Note*. ‘++’: Good growth; ‘+’: Growth; ‘+(w)’: Poor growth; ‘−’: No growth.

### Utilization of various sugars by the LAB strains

The results for carbohydrate fermentation by the LAB strains are shown in Table 3. All the isolated LAB strains were found to utilize glucose, arabinose and maltose. Xylose and trehalose were not utilized by *L. plantarum* TEZU272, whereas, galactose was not utilized by *P. pentosaceus* TEZU427. Cellobiose was not utilized by *L. casei* TEZU309, *P. pentosaceus* TEZU451 and *L. casei* TEZU374. Mannose, on the other hand was not utilized by *L. casei* TEZU309, *L. casei* TEZU374 and *P. pentosaceus* TEZU482. The utilization of sucrose and salicin varied among all the strains. Lactose was however not utilized by *L. casei* TEZU309, *L. casei* TEZU468, *P. pentosaceus* TEZU451, *P. pentosaceus* TEZU427 and *P. pentosaceus* TEZU482. Raffinose, sorbitol and dulcitol were scarcely utilized by all the LAB strains.

**Table 3.** Carbohydrate fermentation profile of the strains

Strain name	Carbohydrates													
	G	L	M	S	X	Mn	T	Ga	R	A	S	So	D	C
* L. casei* TEZU309	++	−	+	−	+(w)	−	++	+(w)	−	+(w)	−	−	++	−
* L. pentosus* TEZU174	++	+(w)	++	++	+	++	+(w)	++	−	++	++	+(w)	−	++
* L. casei* TEZU468	++	−	+(w)	−	+(w)	++	++	−	−	+(w)	−	−	−	+(w)
* P. pentosaceus* TEZU199	++	+(w)	+	−	++	++	++	++	−	++	++	−	−	++
* P. pentosaceus* TEZU451	++	−	++	−	+(w)	−	++	++	−	+(w)	−	−	−	+(w)
* P. pentosaceus* TEZU213	++	+(w)	+	−	++	++	++	++	−	++	++	−	−	++
*L .plantarum* TEZU272	++	++	++	++	−	+(w)	−	+(w)	−	+(w)	−	−	−	++
* P. pentosaceus* TEZU427	++	−	++	−	++	++	++	+	−	++	−	+	−	++
* P. pentosaceus* TEZU481	++	−	++	−	+(w)	−	++	++	−	+(w)	+(w)	−	++	+(w)
* P. pentosaceus* TEZU482	++	−	++	−	+	++	++	+	−	+(w)	−	−	−	−
* P. pentosaceus* TEZU486	++	−	++	−	+	++	++	+(w)	−	+(w)	−	−	++	+(w)
* L. casei* TEZU262	++	++	++	+(w)	++	++	++	++	−	++	++	+(w)	−	++
* P. pentosaceus* TEZU410	++	++	++	++	++	++	++	+	+	++	−	+	+	+
* L. casei* TEZU374	++	+(w)	++	−	+(w)	−	++	++	−	+(w)	−	−	−	−

*Note* 1. G – glucose; L – lactose; M – maltose; S- sucrose; X – xylose; Mn – mannose; T – trehalose; Ga – galactose; R – raffinose; A – arabinose; S – salicin; So – sorbitol; D – dulcitol; C - cellobiose.

*Note* 2. ‘++’: Good growth; ‘+’: Growth; ‘+(w)’: Poor growth; ‘−’: No growth.

### Tolerance of the LAB strains to acid and bile salts

Acid tolerance of the LAB strains at two different pH levels are shown in Table 4. At the pH of 2.5 high resistances was shown by the strains *L. pentosus* TEZU174, *P. pentosaceus* TEZU199, *P. pentosaceus* TEZU199, *L. plantarum* TEZU272, *L. casei* TEZU262, *P. pentosaceus* TEZU410 and *L. casei* TEZU374. *L. pentosus* TEZU174 could maintain a count of 4 log c.f.u. ml^−1^ till 48 h. At the pH of 1.5, *L. pentosus* TEZU174 and *P. pentosaceus* TEZU199 could survive till the fourth hour with counts of 5 log c.f.u. ml^−1^. The results for tolerance of the LAB strains to bile salt are shown in Table 5. It was seen that most of LAB strains were highly tolerant against bile salt. Except *L. casei* TEZU262 and *P. pentosaceus* TEZU410, all the strains could maintain a count of above 7 log c.f.u. ml^−1^ up to a final concentration of 15 % bile salt.

**Table 4.** Acid tolerance of strains at two different pH levels

Strain name	Change in count (log c.f.u. ml^−1^) with time															
	pH 2.5											pH 1.5				
	0 h	1 h	2 h	3 h	4 h	5 h	6 h	7 h	8 h	24 h	48 h	0 h	1 h	2 h	3 h	4 h
* L. casei* TEZU309	9.30	8.90	8.43	8.26	5.95	5.00	5.00	5.00	5.00	0.00	0.00	9.30	7.00	0.00	0.00	0.00
* L. pentosus* TEZU174	8.30	8.03	7.94	7.92	7.77	7.67	7.57	7.08	6.60	5.78	4.00	8.30	7.00	6.48	5.16	5.00
* L. casei* TEZU468	9.48	6.00	0.00	0.00	0.00	0.00	0.00	0.00	0.00	0.00	0.00	9.48	0.00	0.00	0.00	0.00
* P. pentosaceus* TEZU199	8.60	7.99	7.45	7.20	7.15	7.18	6.60	5.78	5.60	0.00	0.00	8.60	7.85	6.56	5.64	5.00
* P. pentosaceus* TEZU451	9.48	7.30	0.00	0.00	0.00	0.00	0.00	0.00	0.00	0.00	0.00	9.48	0.00	0.00	0.00	0.00
* P. pentosaceus* TEZU213	8.45	8.39	8.22	8.16	8.04	7.84	7.75	7.70	7.48	0.00	0.00	8.45	0.00	0.00	0.00	0.00
*L .plantarum* TEZU272	9.60	9.60	9.48	9.48	9.47	9.40	9.40	9.08	9.04	0.00	0.00	9.60	8.25	7.36	7.00	0.00
* P. pentosaceus* TEZU427	9.60	9.30	6.00	0.00	0.00	0.00	0.00	0.00	0.00	0.00	0.00	9.60	0.00	0.00	0.00	0.00
* P. pentosaceus* TEZU481	9.04	5.48	5.00	0.00	0.00	0.00	0.00	0.00	0.00	0.00	0.00	9.04	0.00	0.00	0.00	0.00
* P. pentosaceus* TEZU482	9.30	7.90	7.90	0.00	0.00	0.00	0.00	0.00	0.00	0.00	0.00	9.30	0.00	0.00	0.00	0.00
* P. pentosaceus* TEZU486	7.48	7.00	6.00	0.00	0.00	0.00	0.00	0.00	0.00	0.00	0.00	7.48	0.00	0.00	0.00	0.00
* L. casei* TEZU262	9.07	9.02	8.59	8.54	8.22	7.95	7.52	7.11	7.08	0.00	0.00	9.07	6.29	5.00	0.00	0.00
* P. pentosaceus* TEZU410	9.60	9.40	9.30	8.04	7.60	7.30	7.30	7.00	7.00	4.30	0.00	9.60	9.60	7.11	7.00	0.00
* L. casei* TEZU374	9.60	9.60	9.48	9.48	9.45	9.40	9.30	9.30	9.00	5.08	0.00	9.60	0.00	0.00	0.00	0.00

**Table 5.** Tolerance of the LAB strains to bile salt

Strain name	log c.f.u. ml^−1^ in different bile salt concentrations			
	8 %	10 %	12 %	14 %
* L. casei* TEZU309	7.80	7.56	7.36	6.95
* L. pentosus* TEZU174	7.89	7.83	7.83	7.81
* L. casei* TEZU468	7.86	7.79	7.77	7.52
* P. pentosaceus* TEZU199	7.28	7.26	6.95	6.70
* P. pentosaceus* TEZU451	7.70	7.67	7.15	6.78
* P. pentosaceus* TEZU213	7.30	7.04	6.90	6.90
* L. plantarum* TEZU272	7.77	7.76	7.72	7.70
* P. pentosaceus* TEZU427	7.91	7.83	7.70	7.28
* P. pentosaceus* TEZU481	8.06	7.90	7.60	7.11
* P. pentosaceus* TEZU482	7.99	7.86	7.53	7.30
* P. pentosaceus* TEZU486	7.86	7.83	7.48	7.23
* L. casei* TEZU262	3.00	0.00	0.00	0.00
* P. pentosaceus* TEZU410	7.95	4.73	4.64	4.41
* L. casei* TEZU374	7.40	7.26	7.20	7.20

*Note*. Counts after 24 h of growth in the presence of bile salts.

### Antibiotic susceptibility of the LAB strains

The antibiotic resistance test of LAB strains was performed and it is important to be assessed in order to limit the transmission of antibiotic resistance genes to unrelated pathogenic or opportunistic bacteria. Based on the zones of inhibition, the LAB strains were designated as resistant (R), intermediate (I) and susceptible (S) as per Clinical and Laboratory Standards Institute guidelines [32]. Here, the resistant category includes the isolates which are not inhibited by the usually achievable concentrations of the antibiotic; the intermediate category includes those isolates where the antibiotics usually approach attainable MICs in tissue and blood, while the susceptible category implies those isolates which are inhibited by the usually achievable concentrations of antibiotics. The results for this test are shown in Table 6, and it was observed that all the studied LAB strains were susceptible towards the majority of the tested antibiotics in varying degrees.

**Table 6.** Susceptibility of the LAB strains to different antibiotics

Strain name	Zone of inhibition (mm)											
	AM10	C30	CIP5	E15	P10	GM50	K30	RA30	S25	TE10	VA10	LZD30
* L. casei* TEZU309	29(S)	32(S)	12(R)	34(S)	30(S)	18(S)	0(R)	27(S)	0(R)	18(I)	10(I)	30(S)
* L. pentosus* TEZU174	34(S)	35(S)	14(R)	35(S)	35(S)	22(S)	0(R)	30(S)	16(S)	27(S)	0(R)	37(S)
* L. casei* TEZU468	26(S)	37(S)	12(R)	34(S)	30(S)	24(S)	0(R)	28(S)	13(I)	27(S)	0(R)	37(S)
* P. pentosaceus* TEZU199	24(S)	32(S)	11(R)	31(S)	26(S)	15(S)	0(R)	29(S)	0(R)	23(S)	0(R)	34(S)
* P. pentosaceus* TEZU451	28(S)	35(S)	13(R)	32(S)	26(S)	20(S)	0(R)	31(S)	11(I)	26(S)	10(I)	35(S)
* P. pentosaceus* TEZU213	22(S)	24(S)	12(R)	32(S)	30(S)	21(S)	0(R)	30(S)	10(R)	24(S)	0(R)	35(S)
* L. plantarum* TEZU272	36(S)	38(S)	17(I)	37(S)	35(S)	26(S)	0(R)	32(S)	13(I)	28(S)	0(R)	39(S)
* P. pentosaceus* TEZU427	22(S)	32(S)	12(R)	32(S)	24(S)	19(S)	0(R)	25(S)	10(R)	26(S)	0(R)	32(S)
* P. pentosaceus* TEZU481	23(S)	32(S)	10(R)	30(S)	25(S)	19(S)	0(R)	28(S)	11(I)	23(S)	0(R)	34(S)
* P. pentosaceus* TEZU482	24(S)	32(S)	10(R)	31(S)	28(S)	19(S)	0(R)	28(S)	10(R)	24(S)	10(I)	33(S)
* P. pentosaceus* TEZU486	24(S)	32(S)	11(R)	31(S)	28(S)	18(S)	0(R)	28(S)	10(R)	22(S)	10(I)	34(S)
* L. casei* TEZU262	25(S)	28(S)	33(S)	30(S)	31(S)	24(S)	0(R)	31(S)	16(S)	33(S)	0(R)	38(S)
* P. pentosaceus* TEZU410	24(S)	36(S)	12(R)	32(S)	30(S)	20(S)	0(R)	28(S)	10(R)	25(S)	13(S)	35(S)
* L. casei* TEZU374	23(S)	28(S)	17(I)	30(S)	31(S)	19(S)	0(R)	29(S)	12(I)	29(S)	0(R)	31(S)

*Note*. AM10: Ampicillin (10 mcg); C30 - Chloramphenicol (30 mcg); CIP5: Ciprofloxacin (5 mcg); E15: Erythromycin (15 mcg); P10: Penicillin (10 mcg); GM50: Gentamycin (50 mcg); K30: Kanamycin (30 mcg); RA30: Rifampicin (30 mcg); S25: Streptomycin (25 mcg); TE10: Tetracycline (10 mcg); VA10: Vancomycin (10 mcg); LZD30: Linezolid (30 mcg).

### Antibiosis activity of the LAB strains

**Fig. 2.**
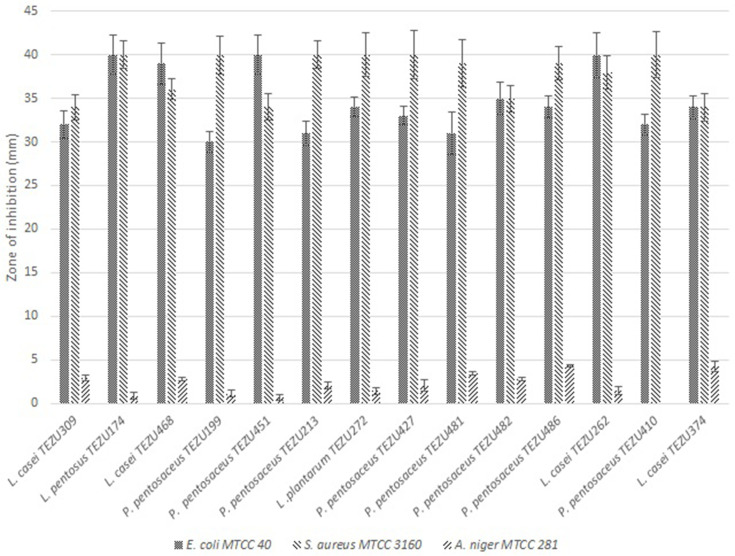
Antibiosis activity of the LAB strains against *E. coli* MTCC 40, *S. aureus* MTCC 3160 and *A. niger* MTCC 281.

The results for antibiosis activity of the LAB strains are illustrated in Fig. 2. It was observed that all the LAB strains were highly antagonist against both *E. coli* and *S. aureus *. In both the cases, the zones of inhibition were above 30 mm, and some of the strains also exhibited zones of inhibition up to 40 mm. In the case of *A. niger*, *P. pentosaceus* TEZU486 and *L. casei* TEZU374 showed the highest inhibition of growth, while all the other strains exhibited zones below 3 mm.

### Antioxidant activity of the LAB strains

**Fig. 3.**
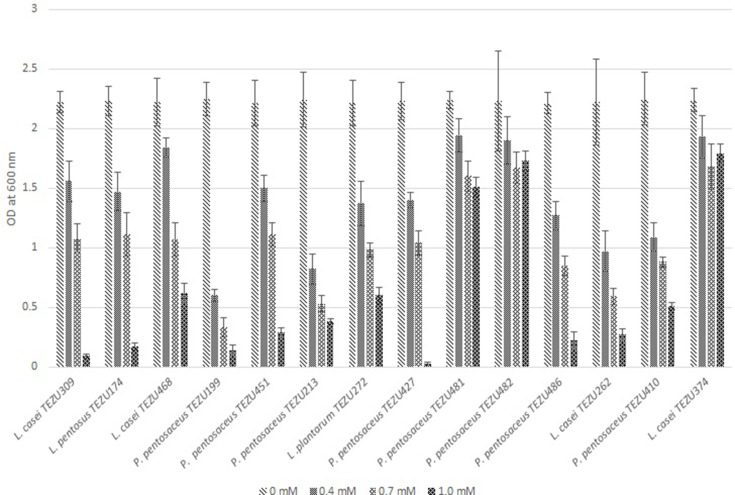
Resistance of the LAB strains to different concentrations (0.0 mM, 0.4 mM, 0.7 mM and 1.0 mM) of H_2_O_2_.

**Fig. 4.**
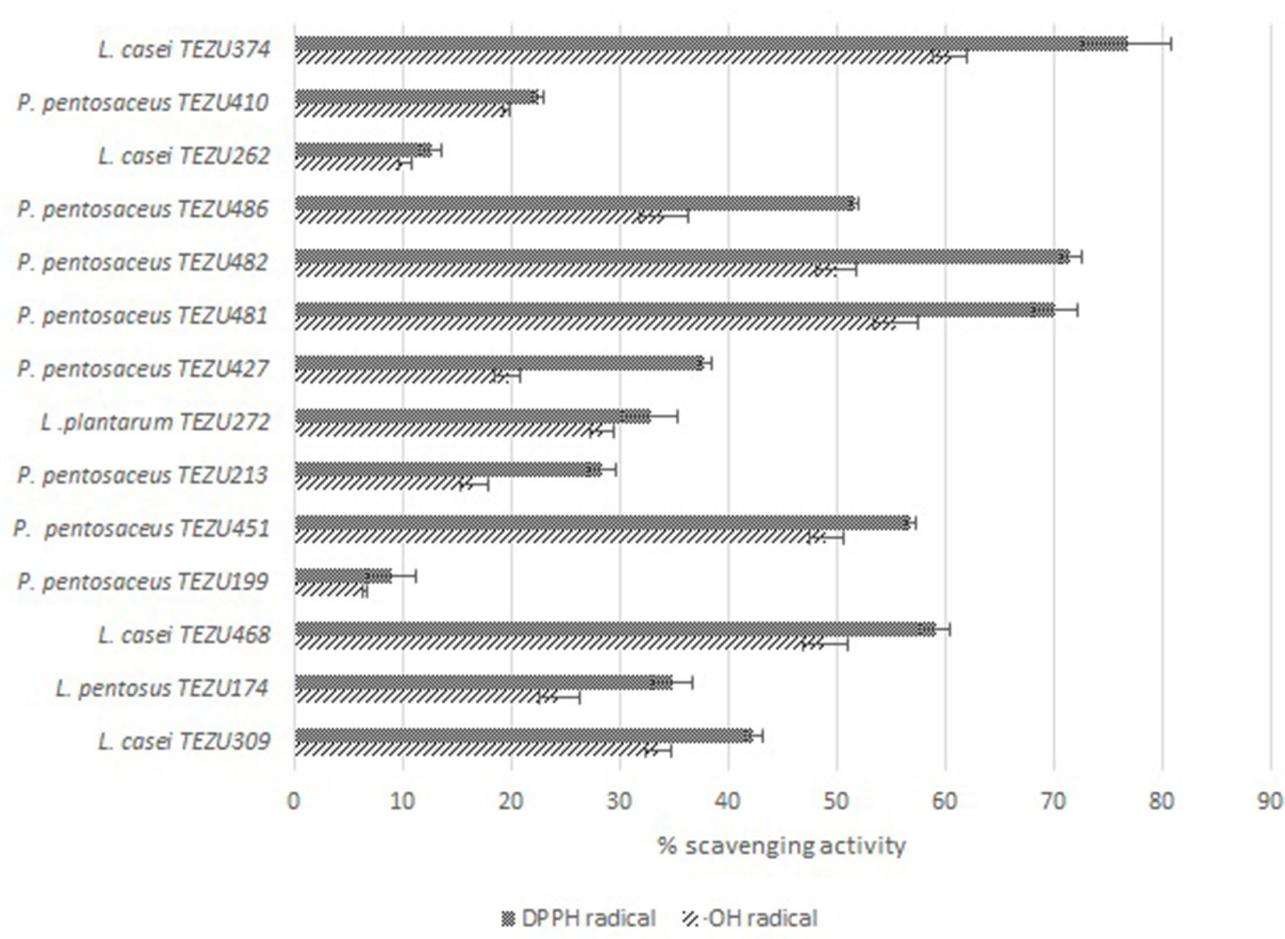
Radical (·OH and DPPH) scavenging activity of the LAB strains.

The antioxidant activity of the LAB strains were assessed in terms of resistance to H_2_O_2_ and scavenging of ·OH and DPPH, and are shown in Figs 3 and 4, respectively. Encouraging results were shown by the strains *P. pentosaceus* TEZU481, *P. pentosaceus* TEZU482 and *L. casei* TEZU374, which maintained OD of above 1 even at concentration of 1.0 mM H_2_O_2_. High ·OH radical scavenging activity was shown by *L. casei* TEZU468 (48.96 %), *P. pentosaceus* TEZU451 (49.07 %), *P. pentosaceus* TEZU481 (55.41 %), *P. pentosaceus* TEZU482 (50.04 %) and *L. casei* TEZU374 (60.54 %). In the DPPH radical scavenging activity also it was observed that that strains *L. casei* TEZU468, *P. pentosaceus* TEZU451, *P. pentosaceus* TEZU481, *P. pentosaceus* TEZU482, *P. pentosaceus* TEZU486 and *L. casei* TEZU374 exhibited activity above 50 %.

### Cellular aggregation and MATS

**Fig. 5.**
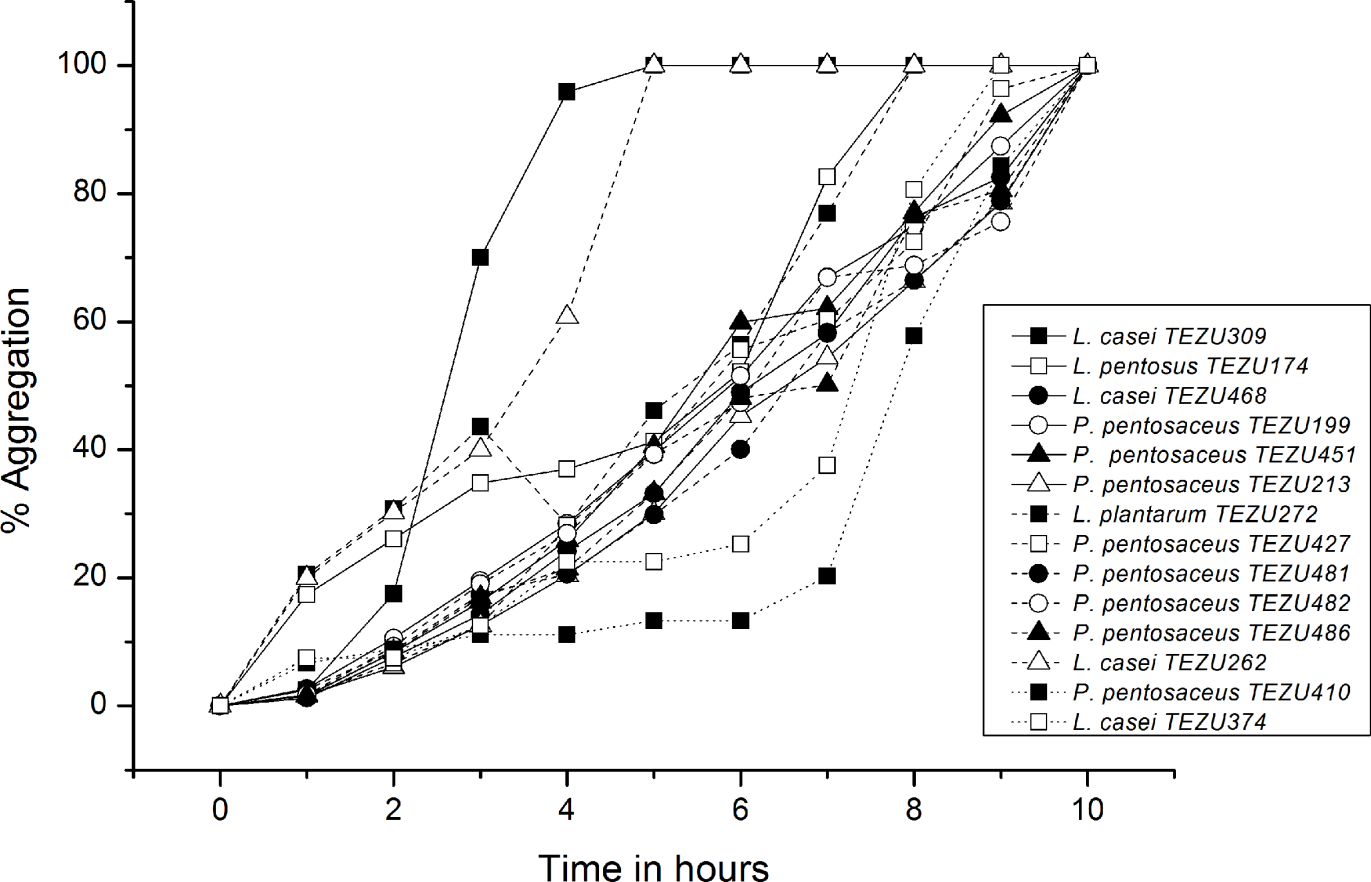
Cellular aggregation of the LAB strains.

**Fig. 6.**
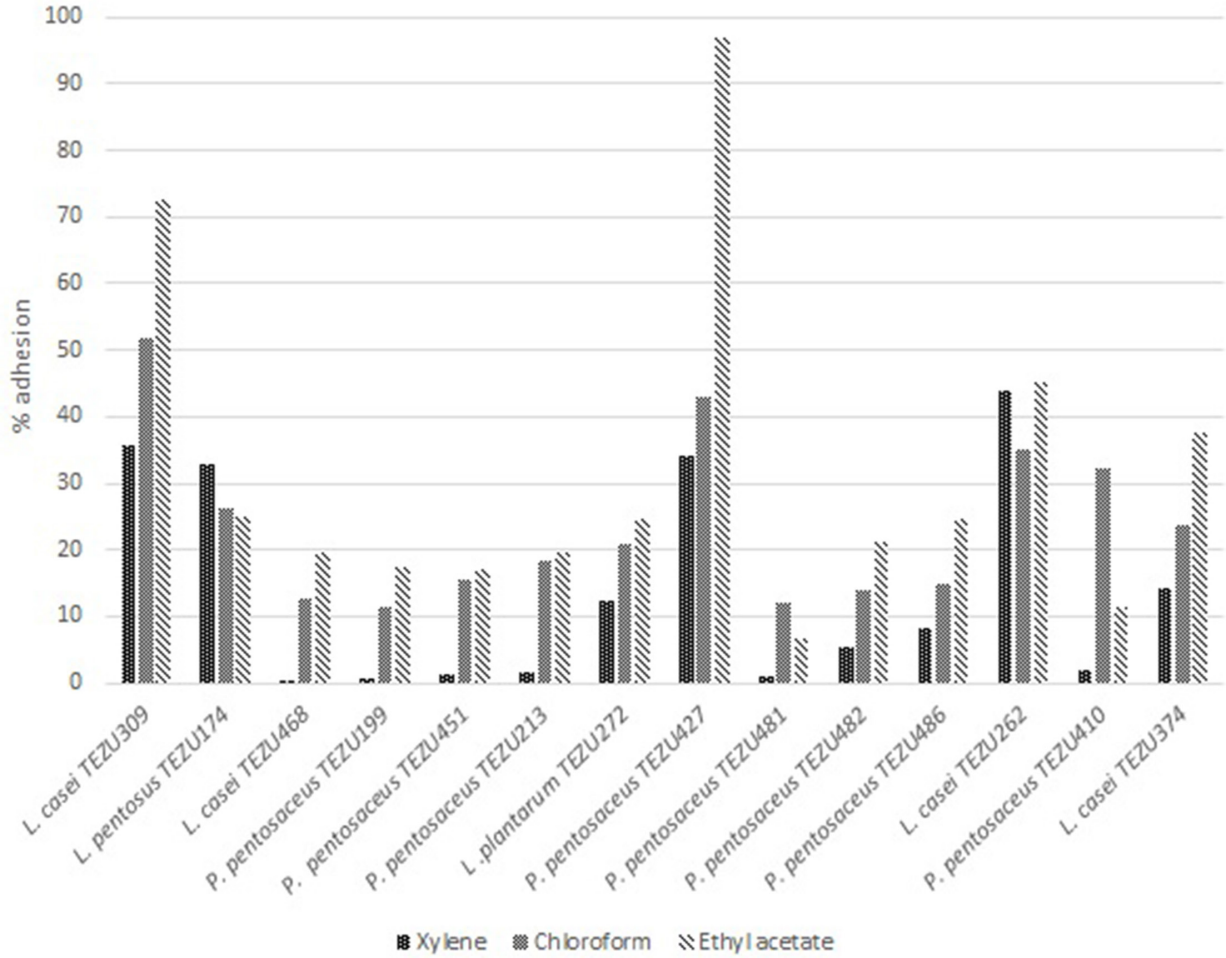
Adhesion of the LAB strains to different solvents after 1 h incubation.

The results for cellular aggregation of the strains are presented in Fig. 5. It was observed that *L. casei* TEZU309 and *L. casei* TEZU262 could obtain 100 % aggregation within 5 h of incubation. *L. pentosus* TEZU174 and *L. plantarum* TEZU272 could attain 100 % aggregation within the eighth h, while the rest of the strains attained the same on the tenth h. Adhesion of the strains to different solvents after 1 h incubation is shown in Fig. 6. It was observed that the percentage of adhesion for all the strains was more in ethyl acetate, followed by chloroform and xylene. The highest percentage of adhesion was shown by *L. casei* TEZU309, *L. pentosus* TEZU174, *P. pentosaceus* TEZU427, *L. casei* TEZU262 and *L.casei* TEZU374.

## Discussions

The *L. casei* group currently consists of the species *L. casei *, *L. paracasei* and *L. rhamnosus *. The species diversity of *L. casei* in regards to its ecological versatility and genome evolution has also been recently evidenced by comparative genomic analyses [33]. Several strains of *L. casei *, have already been identified with potential probiotic traits and satisfying stringent technological performances [34]. *L. casei* has also been used successfully as a probiotic in a number of commercial fermented food products [35]. The species *L. pentosus* is considered to be safe and suitable for the Qualified Presumption of Safety approach by the European Food Safety Authority [36]. It is a versatile candidate for fermentation as it can ferment hexoses using the Embden–Meyerhoff–Parnas pathway and pentoses using the phosphoketolase pathway [37]. It has been shown to inhibit the growth of *Helicobacter pylori* and other pathogens such as *E. coli *, *Salmonella *, *A. niger*, *Aspergillus oryzae*, *Streptococcus pneumonia* and influenza virus [38]. *L. plantarum* is a versatile organism commonly encountered in dairy, meat and plants products, as well as in the gastrointestinal (GI) tract of humans and animals. It has high occurrence in traditional fermented foods from Korea, China and India. The behaviour of different strains of *L. plantarum* is diverse; some may exhibit resistance to extreme acidic conditions, whereas others can resist high concentration of bile salt. This may be attributed to the presence and absence of different genes encoding production of bacteriocin, exopolysaccharides and genes involved in sugar metabolism which in turn affects the adaptability and survivability [39, 40]. *P. pentosaceus* is a homofermentative probiotic LAB, and is known to prevent cardiovascular diseases, attack of harmful pathogens in the GI and provoke immune reaction [41]. It has been used as a starter culture in meat, vegetables and dairy fermentation and is responsible for characteristic flavour changes and extending the shelf life. It also produces bacteriocins active against a broad spectrum of Gram-positive bacteria like *Listeria monocytogenes *, *S. aureus ,* etc. [42].

All the strains were found to be positive for the production of catalase. It was observed that none of the strains produced gas from glucose. The production of gas from glucose is a characteristic feature of the obligately heterofermentative lactobacilli that ferment hexoses to lactic acid, acetic acid and/or ethanol and carbon dioxide [10]. The production of ammonia from arginine was found to be negative in all the strains. The degradation of l-arginine leads to the formation of ornithine and ammonia. The screening of LAB for the ability to degrade arginine is important in their selection as starter cultures since degradation of arginine is also an indicative of the LAB’s ability to form citrulline and carbamyl phosphate, which are precursors of ethyl carbamate, a carcinogen found in fermented foods and alcoholic beverages [43]. None of the LAB strains showed gelatin hydrolysis activity. Hydrolysis of gelatin is indicative of the proteolytic activity of LAB. Even though LAB have only weak proteolytic action on myofibrillar proteins, however, certain strains actively contribute to the hydrolysis of the sarcoplasmic proteins and decomposition of peptides into amino acids. The amino acids also act as precursors of flavour compounds in the final product. Hence screening for gelatine hydrolysis is important for selection of probiotic LAB strains [44]. The haemolytic activity was also absent in all the strains. Testing for haemolytic activity is a safety prerequisite for the selection of probiotic LAB strain. Absence of haemolytic activity is indicative that the strain is non-pathogenic [45].

The LAB strains were found to survive in adverse conditions of pH, osmotic stress and temperatures. In general, the optimum ranges of external pH required for growth and survival of bacteria are between 4 and 8. In an unbalanced pH state, the normal cellular components are not synthesized and the cell does not divide and grow. In conditions of low pH growth may have stopped, but LAB cells may survive and still be metabolically active. In adverse conditions energy-requiring proton pump are used by the bacteria to either pump protons out of the cell (in low pH) or into the cell (in high pH). Osmotic stress causes a reduction in the amount of water available to a micro-organism. LAB have minimum water activity (*a*_w_) limits, below which they cannot grow. The limiting value of *a*_w_ depends on the type of solute viz. salts like NaCl, KCl etc. and sugars like glucose, sucrose etc. In the case of a hyperosmotic shock (low *a*_w_ outside the cell) resulting in a loss of turgor pressure, the bacteria responds by osmoregulation. The bacteria also raise the levels of internal solute resulting in an increase in internal osmotic pressure. They may also cause certain changes in the membrane phospholipid and fatty acids. Growth of LAB below the optimum growth temperature results in growth cessation and changes in metabolic products and due to delays in enzyme activity and metabolic regulatory processes. Membrane leakage occurs when fluid components become gel-like and prevents the proteins from functioning correctly. The tolerant strains adapt to this situation by increasing the proportion of unsaturated fatty acids, thereby allowing the membrane to retain fluidity and prevent gel formation [46].

The LAB strains also showed good resistance to acidic and bile salt conditions. The transit time for any bacteria in the human GI tract can be from less than 1 to 4 h depending on various conditions. Therefore, their survival in the gastric juice depends on their ability to tolerate low pH. Hence, strains of LAB intended for probiotic purposes should be screened for tolerance to low pH [44]. Weak acids affect the cells’ ability to maintain pH homeostasis, thereby disrupting substrate transport and inhibiting metabolic pathways. However, tolerance may be developed when the microbes are exposed to a mild concentration of a weak acid, and rendering them resistant to a stronger dose [46]. After surviving the acidic conditions of the stomach, the ingested bacteria should be able to tolerate the detergent-like emulsifying effect of the bile salts released into the duodenum. The bacteria may achieve it by hydrolysing the bile with bile salt hydrolase enzymes (BSHs), thereby decreasing their solubility [44].

LAB with functional or probiotic properties should be devoid of transferable resistance or resistance genes against antibiotics, as this poses a risk of transfer to pathogenic bacteria. From the results obtained, it was observed that the studied LAB strains will be affected during antibiotic treatment. Certain degree of resistance displayed towards kanamycin, vancomycin and ciprofloxacin may be due to natural or intrinsic factors, which poses no risk in non-pathogenic bacteria [47]. Moreover, lactobacilli have a high natural resistance to vancomycin due to the presence of highly conserved non-transferable genes in the chromosome [48]. Our results are also similar to the findings of Guo *et al.* [49], who studied the antibiotic resistance of *Lactobacillus* species isolated from traditional dairy products, and found that most of the *L. plantarum* and *L. casei* strains were resistant to vancomycin, kanamycin and ciprofloxacin. They concluded that the resistance was conferred due to the presence of *van*X, *aph*(3″)-III and *gyr*A genes responsible for vancomycin, kanamycin and ciprofloxacin resistance, respectively.

The results for the antagonism effect demonstrated that the LAB strain were active against pathogenic bacteria either via antimicrobial substance production or competitive exclusion, and thus have an impact on the colonic flora. They may produce bacteriocins, low molecular weight metabolites such as hydrogen peroxide, lactic and acetic acid, certain aroma compounds and secondary metabolites. Probiotics have been found to exhibit inhibitory spectrum against many harmful organism like *Salmonella *, *E. coli *, *Clostridium* and *Helicobacter* [50]. The antimicrobial activity shown against the potentially pathogenic Gram-negative and Gram-positive bacteria also indicated that these strains can reduce the number of undesired micro-organisms in fermented food products and make them for human consumption.

Reactive oxygen species (ROS) are produced during passage of food through the GI tract, leading to oxidative damage, which forms a part in the pathogenesis of cancer, cirrhosis, atherosclerosis and other chronic diseases [51]. The antioxidative activity of the LAB strains may thus decrease the risk of accumulation of ROS in the GI tract through mechanisms like ROS scavenging, metal ion chelation, enzyme inhibition, reduction activity and inhibition of ascorbate autoxidation [52]. The important components of their defence against ROS damage are reduced glutathione and antioxidative enzymes superoxide dismutase, glutathione peroxidise and catalase [53].

The adhesion ability of LAB such as hydrophobicity and the production of exopolysaccharides are involved in the modulation of host immune response. Autoaggregation is the ability of clumping of the cells of the same strain to form multicellular aggregates [54]. In order to exert beneficial probiotic effects, LAB need to achieve an adequate mass through aggregation. Autoaggregation correlates adhesion, which is a complex process involving non-specific (hydrophobicity) and specific ligand-receptor mechanisms [55]. Whereas, the MATS technique is used because it helps in determining not only hydrophobicity but also the electron donor or electron receptor character of the cell surface, which is another method to explore adhesion. The results indicate that the isolated strains have moderate adhesion capacity. Certain strains also showed high adherence scores for chloroform, which is a monopolar solvent. This may be due to the basic properties (Lewis base) of the bacterial cell surface, which is again related to the presence of a carboxylic group [56]. Adherence of LAB to mucosal surfaces and intestinal epithelium is related to the cell surface characteristics. The surface properties of LAB play an important role in the adhesion of the bacteria to the gastrointestinal epithelium, which is considered to be a prerequisite for the exclusion of enteropathogenic bacteria or immunomodulation of the host [57]. The peptidoglycan layer of the cell wall of LAB is covered by a variety of substances like lipoteichoic acids, neutral and acidic polysaccharides, and surface proteins [57] and their adhesive properties include different features like passive forces, electrostatic interactions and hydrophobic steric forces [54].

Thus, fundamental growth and metabolic activity studies of *L. casei* (three strains), *L. pentosus* (one strain), *L. plantarum* (one strain) and *P. pentosaceus* (eight strains) isolated from rice beer and starter cakes prepared in Assam revealed that the strains could grow at low pH, presence of bile salts, high osmotic pressure and variable temperatures. They were susceptible towards most of the studied antibiotics and were antagonist against both *E. coli* and *S. aureus *. All of them also evinced antioxidant activity and cell surface hydrophobicity. These LAB strains exhibited properties, which are characteristics of probiotic bacteria, and this study may provide wider application avenues for these strains to be used as starter cultures.
